# AI-Driven Integration of Deep Learning With Lung Imaging, Functional Analysis, and Blood Gas Metrics for Perioperative Hypoxemia Prediction

**DOI:** 10.2196/73995

**Published:** 2025-08-22

**Authors:** Kecheng Huang, Chujun Wu, Rongpeng Pi, Jieyu Fang

**Affiliations:** 1 Department of Anesthesiology Guangxi Hospital Division of The First Affiliated Hospital, Sun Yat-sen University Nanning China; 2 Department of Anesthesiology Sun Yat-sen Memorial Hospital Guangzhou China; 3 Department of Anesthesiology The First Affiliated Hospital, Sun Yat-sen University Guangzhou China

**Keywords:** pneumonia, perioperative, artificial intelligence, hypoxemia, deep learning

## Abstract

This viewpoint article explores the transformative role of artificial intelligence (AI) in predicting perioperative hypoxemia through the integration of deep learning with multimodal clinical data, including lung imaging, pulmonary function tests, and arterial blood gas (ABG) analysis. Perioperative hypoxemia, defined as arterial oxygen partial pressure <60 mmHg or oxygen saturation <90%, poses significant risks of delayed recovery and organ dysfunction. Traditional diagnostic methods such as radiological imaging and ABG analysis often lack integrated predictive accuracy. AI frameworks, particularly convolutional neural networks and hybrid models like TD-CNNLSTM-LungNet, demonstrate exceptional performance in detecting pulmonary inflammation and stratifying hypoxemia risk, achieving up to 96.57% accuracy in pneumonia subtype differentiation and an area under the curve of 0.96 for postoperative hypoxemia prediction. Multimodal AI systems, such as DeepLung-Predict, unify computed tomography scans, pulmonary function tests, and ABG parameters to enhance predictive precision, surpassing conventional methods by 22%. However, challenges persist, including dataset heterogeneity, model interpretability, and clinical workflow integration. Future directions emphasize multicenter validation, explainable AI frameworks, and pragmatic trials to ensure equitable and reliable deployment. This AI-driven approach not only optimizes resource allocation but also mitigates financial burdens on health care systems by enabling early interventions and reducing intensive care unit admission risks.

## Introduction

Pulmonary inflammation, encompassing pneumonia, COVID-19 sequelae, and chronic obstructive pulmonary disease, continues to be a predominant cause of perioperative complications, particularly hypoxemia [1-4]. Hypoxemia, defined as arterial oxygen partial pressure (PaO_2_) <60 mmHg or oxygen saturation (SpO_2_) <90%, poses considerable risks during the perioperative period, including delayed recovery and organ dysfunction [5]. Traditional diagnostic approaches, such as radiological imaging, pulmonary function tests (PFTs), and arterial blood gas (ABG) analysis, are mutually independent and frequently lack integrated predictive accuracy [6,7]. Artificial intelligence (AI), particularly deep learning, has emerged as a revolutionary tool for the early detection of pulmonary inflammation and proactive risk stratification [8]. This article examines the recent progress in AI-driven analysis of radiological imaging, preoperative PFTs, and ABG parameters for predicting perioperative hypoxemia, while addressing challenges and future directions.

Emerging evidence suggests that preoperative PFTs and ABG parameters serve as independent predictors of postoperative hypoxemia, yet clinical decision-making in this context fundamentally incorporates clinician expertise derived from longitudinal patient management experience. Physicians may demonstrate substantial interclinician variability in prognostic estimations when relying on experiential clinical judgment, potentially leading to significant discrepancies in risk stratification outcomes.

With AI evolution in the medical field, it is highly necessary in clinical practice to integrate the results of these preoperative examinations to predict postoperative hypoxemia. Current clinical practice necessitates the integration of PFTs and ABG, computed tomography (CT) images, body surface area, and other factors to enhance predictive accuracy. This highlights the need for AI-driven, multivariate, predictive modeling that synergistically combines multifactorial determinants of postoperative respiratory function. Such an advanced computational approach promises to optimize risk stratification while conserving critical health care resources through precision medicine implementation.

## AI in Pulmonary Inflammation Recognition: Technological Foundations

### Deep Learning for Lung Imaging Analysis

Modern AI frameworks have revolutionized the detection and classification of pulmonary inflammation through advanced analysis of radiographs, CT scans, and dynamic ultrasound imaging. State-of-the-art convolutional neural networks (CNNs), such as Mask R-CNN, leverage hierarchical feature extraction to achieve unprecedented diagnostic precision. By integrating ResNet50/101 backbones with feature pyramid networks, Mask R-CNN attains 88.3% classification accuracy (95% CI 86.4-89.8) and 83.13% regression precision (intersection over union ≥0.5) in localizing pneumonia lesions on chest X-rays. This architecture uses region proposal networks to enhance sensitivity for subcentimeter ground-glass opacities, outperforming traditional computer-aided diagnosis systems in multicenter trials [9].

Hybrid models have further bridged the gap between spatial and temporal diagnostics. The TD-CNNLSTM-LungNet framework synergizes 3D CNNs for volumetric pattern recognition and bidirectional long short-term memory networks to decode temporal variations in lung ultrasound videos [10]. Evaluated on 1243 cases, this model achieved 96.57% accuracy in differentiating pneumonia subtypes (eg, viral versus bacterial) by analyzing dynamic features such as air bronchograms and consolidations. Its superior performance is evidenced by an area under the curve (AUC) of 0.983 (95% CI 0.972-0.991) in distinguishing COVID-19–related interstitial patterns from bacterial lobar infiltrates, significantly surpassing radiologist consensus (AUC=0.872, *P*=.003) [10].

AI systems now decode pathognomonic imaging biomarkers with submillimeter precision. For instance, VGG-Pneumonia v2.0 [11], a transfer learning–enhanced model, enables granular preoperative risk stratification. Attention mechanisms in DenseNet-121/Inception-v3 ensembles [12] have improved early detection of interstitial pneumonia to 91.4% sensitivity (κ=0.86) by mapping septal thickening patterns. Crossmodal fusion techniques also correlate CT-derived honeycombing scores with spirometry data to predict disease progression (*r*=0.79, *P*<.001) [13].

### Integration of Pulmonary Function Metrics

Preoperative PFTs serve as critical tools for evaluating respiratory reserve and predicting postoperative outcomes in surgical patients. Key parameters such as forced expiratory volume in 1 second (FEV_1_), forced vital capacity (FVC), and diffusion capacity for carbon monoxide (DLCO) collectively offer multidimensional insights into pulmonary mechanics and gas exchange efficiency [14,15]. FEV_1_ quantifies the maximum air volume forcibly exhaled within the first second, reflecting airway patency and obstructive patterns. FVC measures total expiratory volume after maximal inspiration, indicating restrictive lung disease when reduced [16,17]. The FEV_1_/FVC ratio further differentiates between obstructive and restrictive pathologies, with values below 0.7 typically suggesting airflow limitation [15]. DLCO evaluates alveolar-capillary membrane integrity through carbon monoxide diffusion capacity, directly correlating with postoperative oxygenation capacity [14,18].

Clinical evidence demonstrates these metrics’ prognostic significance. In cardiac surgery populations, reduced FEV_1_ (<70% predicted) and FVC (<80% predicted) independently associate with prolonged mechanical ventilation and increased pulmonary complications [14]. For patients with thoracic oncology, a DLCO <60% predicted is associated with 3.2-fold higher risk of postoperative respiratory failure compared to normal values [18]. The combined assessment proves particularly valuable in high-risk cohorts such as severe scoliosis patients, where 36.7% exhibited mild pulmonary dysfunction detectable through PFT abnormalities [15].

Emerging methodologies enhance predictive accuracy through computational models. Adaptive neuro-fuzzy inference systems now achieve 89% correlation between spirometric measurements and actual postoperative lung function [16]. Nevertheless, standardized protocols remain debated, particularly regarding universal testing versus selective application based on surgical type and patient comorbidities [14,15]. Current practice guidelines recommend preoperative PFTs for patients with unexplained dyspnea, smokers, and those undergoing thoracic or major abdominal procedures to stratify perioperative risks and optimize respiratory management [14,17].

### Blood Gas Analysis and Hypoxemia Prediction

ABG parameters—including PaO_2_, partial pressure of carbon dioxide, bicarbonate, and the PaO_2_/fraction of inspired oxygen ratio—serve as fundamental diagnostic biomarkers for hypoxemia and respiratory insufficiency [19]. Contemporary AI models use machine learning frameworks to analyze these biomarkers for perioperative oxygen demand forecasting [20]. As demonstrated by a multicenter, prospective cohort study, preoperative ABG trends in pH, partial pressure of carbon dioxide, and alveolar-arterial gradients can predict severe hypoxemia (PaO_2_ <50 mmHg) with high accuracy. Specifically, XGBoost algorithms trained on serial ABG measurements achieved 89% sensitivity and 92% specificity in identifying patients at risk of critical oxygen desaturation within the first postoperative hour [20].

The integration of real-time biosensor data further optimizes predictive performance. Adaptive AI systems now synchronize intraoperative pulse oximetry (SpO_2_) waveforms with ventilator controls, reducing hypoxemic events through dynamic intervention [21]. A 2022 study demonstrated that the results of a deep neural network using photoplethysmography signals to predict the severity of hypoxemia in hospitalized patients showed an accuracy rate of 96.5% in determining 3 severity categories, with a Cohen κ score of 0.79. This approach has the potential to help patients benefit from automatic and faster clinical decision support systems, thereby addressing the severity of hypoxemia [22].

## Multimodal AI Systems for Perioperative Risk Stratification

### Fusion of Imaging, Functional, and Biochemical Data

Emerging platforms such as DeepLung-Predict integrate CT scans, PFTs, and ABG parameters into a unified predictive framework. For instance, deep lung parenchyma enhancement, a multimodal AI tool, filters out nonparenchymal CT features to accentuate inflammation-induced fibrosis while concurrently analyzing DLCO and PaO_2_ to assess oxygenation capacity. In a 2024 trial, DeepLung-Predict attained an AUC of 0.96 for predicting postoperative hypoxemia in patients with lung resection, surpassing conventional methods by 22% [23].

### Dynamic Risk Monitoring

The capacity of AI to process real-time data facilitates dynamic risk assessment. For example, recurrent neural networks undertake the analysis of intraoperative SpO_2_, end-tidal carbon dioxide, and ventilator waveforms to predict hypoxemic crises several minutes prior to clinical manifestation [24]. Frequent assessment of the severity of illness for hospitalized patients is essential in clinical settings to prevent outcomes such as in-hospital mortality and unplanned admission to the intensive care unit. Classical severity scores have typically been developed using relatively few patient features. Recently, deep learning–based models demonstrated better individualized risk assessments compared to classic risk scores, thanks to the use of aggregated and more heterogeneous data sources for dynamic risk prediction [25]. 

## Case Study: COVID-19 Recovery and Surgical Planning

Patients who have recovered from COVID-19 often exhibit residual lung fibrosis, increasing hypoxemia risk. AI systems like AI-ADS integrate postinfection CT scans (quantifying fibrosis volume) with preoperative PFTs to guide surgical timing [26]. An AI-driven radiographic analysis system demonstrated enhanced predictive accuracy in stratifying patients hospitalized with COVID-19 requiring mechanical ventilation support during critical disease progression. Following rigorous prospective evaluation and external validation, this computational framework may provide clinical decision support for respiratory failure risk stratification and facilitate timely therapeutic interventions during acute phases of COVID-19 infection [27].

The accuracy of different AI models in predicting hypoxemia is shown in Table 1. A comparative analysis evaluating the predictive efficacy of conventional clinical assessment tools (including pulse oximetry trends and risk stratification scores) versus machine learning algorithms (specifically logistic regression and neural network models) in anticipating perioperative hypoxemia events is systematically detailed in Table 2.

**Table 1:** Performance comparison of artificial intelligence models in hypoxemia prediction.

Model	Data type	Accuracy (%)	AUC^a^	Key strength
Mask R-CNN	Chest X-rays	88.3	0.91	High lesion localization precision (IoU^b^ ≥0.5) in multicenter trials with standardized imaging protocols.
TD-CNNLSTM-LungNet	Lung ultrasound videos	96.57	0.983	Superior spatiotemporal feature analysis validated on 1243 cases across 5 hospital systems.
DeepLung-Predict	CT^c^, PFTs^d^, and ABG^e^	—^f^	0.96	Multimodal fusion, 22% improvement versus conventional methods in patients with lung resection (n=412) [23].
XGBoost (ABG trends)	Serial ABG metrics	89 (sensitivity)	0.89	Early desaturation prediction (PaO_2_^g^ <50 mmHg) within 1 h postoperative in cardiac surgery cohort.
Res-SE-ConvNet	Photoplethysmography	96.5	—	Real-time severity categorization (κ=0.79) using ICU^h^ biosensors at 125 Hz sampling rate.

^a^AUC: area under the receiver operating characteristic curve.

^b^IoU: intersection over union for lesion localization.

^c^CT: computed tomography.

^d^PFT: pulmonary function test.

^e^ABG: arterial blood gas.

^f^Data not available.

^g^PaO_2_: partial pressure of oxygen.

^h^ICU: intensive care unit.

**Table 2:** Comparative performance of traditional versus artificial intelligence (AI)–driven methods for predicting perioperative hypoxemia.

Parameter	Traditional methods	AI-driven models
Diagnostic modalities	CT^a^, PFTs^b^, ABG^c^ (isolated)	Integrated CT + PFTs + ABG + BSA^d^
Sensitivity (%)	68-75	89-94
Specificity (%)	72-78	91-96
AUC^e^	0.70-0.76	0.89-0.93
Time to diagnosis	24-48 hours	Real-time (<5 minutes)
Key limitations	Subjective interpretation and fragmented data	Requires large, annotated datasets and computational resources

^a^CT: computed tomography.

^b^PFT: pulmonary function test.

^c^ABG: arterial blood gas.

^d^BSA: body surface area.

^e^AUC: area under the receiver operating characteristic curve.

## Methodological and Translational Challenges

### Dataset Variability and Generalization

While AI models demonstrate high accuracy in controlled settings, their real-world applicability is hindered by dataset heterogeneity. Variations in imaging protocols (eg, CT slice thickness and X-ray exposure parameters), population demographics (eg, underrepresented ethnicities in training cohorts), and annotation inconsistencies (eg, inter-radiologist variability in labeling pneumonia lesions) limit model generalizability. For instance, a 2023 study found that models trained on single-center data experienced a 15%-20% performance drop when validated on external datasets [28]. Federated learning and standardized imaging protocols (eg, Radiological Society of North America guidelines for COVID-19 CTs) are emerging solutions to mitigate these biases [29].

### Validation Methods and Generalizability

A 2023 meta-analysis highlighted that AI models trained on narrow datasets experience a median performance drop of 18% (95% CI 14%-22%) when applied to external cohorts, underscoring the risk of overestimating real-world efficacy [28].

### Interpretability of AI Systems

The “black box” nature of deep learning models remains a barrier to clinical adoption. While attention maps in CNNs (eg, Grad-CAM visualizations [30]) partially elucidate decision-making processes, they lack granular pathophysiological correlations. Hybrid approaches integrating explainable AI frameworks, such as Shapley addictive explanations values or local interpretable model-agnostic explanations, could bridge this gap [31].

### Integration into Clinical Workflows

Operationalizing AI tools requires addressing infrastructural and human factors. Technical hurdles include interoperability with electronic health records and real-time data latency. Clinician acceptance further depends on workflow integration (eg, embedding AI alerts into perioperative dashboards rather than standalone systems). A 2024 survey revealed that 68% of anesthesiologists prioritize AI tools offering actionable recommendations (eg, ventilator adjustments) over raw risk scores [32]. Pilot studies deploying AI algorithm development for COVID-19 recovery planning achieved 92% adherence when coupled with clinician training modules [33].

## Limitations

AI-based models require large-scale validation in real-world clinical scenarios, and further validation in globally representative countries is needed.

Hospital administrators grapple with AI’s infrastructural demands. Legacy systems hinder data integration. Cost barriers also limit adoption, as implementing AI-driven predictive analytics in a mid-sized hospital requires investment in AI-related infrastructure. Strategic partnerships between health care providers and AI developers, coupled with government subsidies, could mitigate these barriers [34].

Ethical dilemmas remain one of the critical issues warranting attention. AI systems trained on biased datasets may perpetuate disparities in treatment recommendations. Moreover, data anonymization failures and crossinstitutional data sharing raise privacy risks, as seen in cases where deidentified patient records were reidentified through AI-driven linkage [35]. Addressing these issues requires robust anonymization techniques, bias-correction algorithms, and legally defined responsibility matrices.

AI models in perioperative care face unique challenges due to high patient heterogeneity and clinical workflow variability. Overfitting—driven by small, homogenous training datasets—remains a critical concern.

Future efforts must prioritize multicenter, diverse datasets to enhance generalizability, develop hybrid interpretable models, and conduct pragmatic trials evaluating workflow integration. Collaborative frameworks involving clinicians, data scientists, and policymakers will be pivotal in realizing AI’s full potential.

## Conclusion

AI has revolutionized the diagnostics of pulmonary inflammation, providing rapid, precise, and scalable solutions. From DeepLung-Predict to self-supervised X-ray systems, these technologies enhance clinical decision-making without supplanting human expertise. Future progress hinges on addressing data deficiencies, improving interpretability, and guaranteeing equitable access. As AI keeps evolving, its role in the management of respiratory diseases and prediction of perioperative hypoxemia will broaden, ultimately improving global health outcomes.

By leveraging the developed large-scale predictive model to predict perioperative hypoxemia, this framework enables the stratification of postoperative intensive care unit admission risks, thereby facilitating early clinical interventions. Such an approach holds significant potential for optimizing the allocation of health care resources and mitigating financial burdens on health care insurance systems.

## Abbreviations

ABG: arterial blood gas
AI: artificial intelligence
AUC: area under the curve
CNN: convolutional neural network
CT: computed tomography
DLCO: diffusion capacity for carbon monoxide
FEV_1_: forced expiratory volume in 1 second
FVC: forced vital capacity
PaO_2_: oxygen partial pressure
PFT: pulmonary function test
SpO_2_: oxygen saturation
